# Experiences in Participatory Surveillance and Community-based Reporting Systems for H5N1 Highly Pathogenic Avian Influenza: A Case Study Approach

**DOI:** 10.1007/s10393-014-0916-0

**Published:** 2014-03-19

**Authors:** Jeffrey C. Mariner, Bryony A. Jones, Saskia Hendrickx, Ihab El Masry, Yilma Jobre, Christine C. Jost

**Affiliations:** 1 International Livestock Research Institute, PO Box 30709, Nairobi, 00100 Kenya; 2 Royal Veterinary College, London, UK; 3 UN Food and Agriculture Organization, Cairo, Egypt; 4 CGIAR Research Program on Climate Change, Agriculture and Food Security (CCAFS), Nairobi, Kenya

**Keywords:** participatory, epidemiology, surveillance, influenza, veterinary, poultry

## Abstract

Participatory surveillance (PS) is the application of participatory rural appraisal methods to the collection of epidemiological information to inform decision-making and action. It was applied in Africa and Asia as part of emergency programs to address the H5N1 highly pathogenic avian influenza (HPAI) pandemic. The approach resulted in markedly increased case detection in countries experiencing HPAI, and a better understanding of the epidemiological situation. Where HPAI was absent and PS was implemented, the method did not result in false positives and contributed to the overall epidemiological assessment that the country was free of disease. It was noted that clarity of surveillance objectives and resulting data needs at the outset was essential to optimize the balance of surveillance methods, size of the program and costs. The quality of training programs and adherence to international guidelines on good PS training practice were important for assuring the competence of PS practitioners. Orientation of senior decision-makers was an important step in assuring effective program management and appropriate use of results. As a problem-solving methodology, PS is best used to rapidly assess situations and inform strategy. Several countries continued PS after the end of projects and went on to apply PS to other health challenges.

## Introduction

Participatory epidemiology (PE) is the use of participatory rural appraisal (PRA) (Chambers 2007) by professionals to understand epidemiological scenarios and develop best-bet assessments of health situations (Catley et al. 2012; Mariner et al. 2011a; Jost et al. 2007; Mariner and Paskin 2000). Participatory rural appraisal is a semi-structured problem-solving methodology that allows experts to learn about traditional knowledge systems, community perceptions, and priorities (Chambers 2007). Participatory surveillance (PS) is the application of PE to on-going surveillance programs, and seeks to strengthen the gathering of epidemiological intelligence to inform decision-making and action (Mariner et al. 2011a; Jost et al. 2007; Mariner and Paskin 2000). These approaches emphasize learning from stakeholders, rather than just extracting data, to improve our understanding of the social and cultural contexts that affect the distribution and dynamics of diseases as well as the suitability of control alternatives (Mariner et al. 2002; Bett et al. 2009). PS is not a static concept. It has evolved over the years and been applied in a number or different ways. Local adaptation and innovation is encouraged.

Participatory surveillance includes active outreach to stakeholders to enhance the access of stakeholders to the system. The approach is built on respect for traditional knowledge and emphasizes co-learning by both practitioners and participants. It assumes that good communication depends on mutual respect and trust. Participatory methods explicitly seek to improve our understanding of livestock systems, in order to enhance interpretation of information and for setting intervention strategies that lead to more constructive action by all stakeholders. They have been found to create incentives for both reporters and surveillance actors in terms of more positive experiences at both the social and technical level (Mariner et al. 2003). Community-based and participatory surveillance methods do not replace conventional surveillance and analytical capacities. They extend the capabilities of the system with additional information and enhance the ownership of data collection activities by communities (Mariner et al. 2002).

Local communities often recognize the principal diseases that affect their livestock and have a rich terminology for distinguishing between them (Catley et al. 2001, 2012). Knowledge systems often include the history and patterns of diseases in an area. For diseases with significant impact, communities often actively track the evolution of current outbreaks and take actions to avoid affected areas and mitigate risk. Participatory surveillance taps into these information networks (Mariner and Paskin 2000).

Participatory rural appraisal is often described as a tool kit of methods that include semi-structured interviews, scoring, ranking, and visual tools like participatory maps and timelines (Chambers 2007). The emphasis is on using flexible approaches that allow the participants to express themselves in their own knowledge system and provide direction to the interview process. This is very different from the structured interview process used in other epidemiological methods, where questions are formulated from the interviewer’s frame of reference.

For many countries the main component of the livestock disease surveillance system is passive reporting—livestock keepers make a report of a disease event to veterinary services—but the data generated is usually not representative of the disease situation due to under reporting, especially from remote, under-served, marginal areas. If there is a control program for a specific disease, then active surveillance may be conducted such as random-sample cross-sectional surveys that collect samples for laboratory analysis. These can be expensive and logistically demanding if a representative sample is to be achieved, and there is often a delay between the survey and reporting of results. On the other hand, PS is an active surveillance method that is usually used for purposive surveillance in high-risk areas for a specific disease of interest, and combines livestock keeper disease descriptions, observations of livestock, and diagnostic sample collection from animals that fit the case definition of the disease of interest. This generally leads to an increase in the number of cases detected, if the disease is present, and an improved description of the local epidemiological situation. It can also be used to provide evidence on absence of a disease in a population (Mariner et al. 2003). Participatory surveillance methods are not intended to be stand-alone methods, but are used to complement existing activities to increase sensitivity and ensure that high risk and marginalized populations are well represented in the overall system (Mariner et al. 2002).

The first application of PRA to epidemiological issues was when community-based animal health programs used it to conduct animal health assessments to inform program design. Participatory surveillance evolved during the global eradication of rinderpest when the tools were adapted to searching for rinderpest outbreaks. This revealed that livestock keepers had a very detailed knowledge of rinderpest epidemiology and distribution (Mariner and Paskin 2000). The PS approach proved its utility by identifying occult foci of disease and providing appropriate intelligence to guide the eradication strategy (Mariner and Roeder 2003). Since that time, PS has been applied for a number of different diseases in a variety of countries, e.g., rinderpest in East Africa (Mariner and Roeder 2003) and Pakistan (Mariner et al. 2003), FMD in Turkey (Admassu 2005) and Africa (Catley et al. 2004), peste des petits ruminants (PPR) in Pakistan (Hussain et al. 2008) and East Africa (Kihu et al. 2012), classical swine fever in Bolivia (Mariner et al. 2011b), and Rift Valley fever in East Africa (Jost et al. 2010). These programs included applications in pastoral, mixed agricultural, peri-urban and intensive systems, and all major food animal species. It has also been used as a tool to contribute to the verification of absence of disease in the final stages of an eradication program, e.g., rinderpest (Mariner et al. 2003).

In 2009, stakeholders active in PE and PS came together to form the Participatory Epidemiology Network for Animal and Public Health (PENAPH). This organization includes all the major international organizations active in animal and public health as well as selected universities and non-governmental organizations. The mission of PENAPH is to facilitate research, build capacity and set standards in PE, as well as to promote information sharing (Mariner et al. 2012; PENAPH 2013).

The aim of this paper is to describe how PS was applied to highly pathogenic avian influenza (HPAI) H5N1 surveillance in Indonesia, Egypt, and sub-Saharan Africa, and to highlight the lessons learned for the future from these case studies. It is important to bear in mind that HPAI in poultry presented new challenges for the PS methodology in terms of field diagnosis and the socio-economics of disease control in a species with low economic value per unit and high population numbers.

## Methods

We describe four examples of the use of PS for HPAI surveillance since 2006. They were selected because they were the largest applications of PS for this purpose and represent different parts of the world, poultry production systems, and epidemiological situations. The co-authors include principle implementers for each of the four examples and unless otherwise noted this is the first peer-reviewed publication documenting the work. For each case, the history, establishment and main characteristics of the PS system, the results that the system achieved and the positive and negative lessons of the experience are described. The raw material for lessons learned was drawn from the discussions of program managers and national and international stakeholders during training workshops and at national and international meetings. The co-authors have shared this information and the overall lessons learnt reflect the consensus of the co-authors on the key, relevant experience for future programs.

## Results

### Example 1: Indonesia

#### Background

H5N1 HPAI was first detected in Indonesia on the island of Java in December 2003 (Lam et al. 2008), and was reported to the OIE in February 2004 (Azhar et al. 2010). It is hypothesized that the virus was introduced to Java and spread from there to other islands (Lam et al. 2008). At the outset of the PS program, the distribution and extent of HPAI in Indonesia was largely unknown. Indonesia has over 300 million backyard poultry, and vast commercial poultry industry estimated to hold an annualized population of 900 million birds (ILRI 2012). The veterinary services of Indonesia are highly decentralized with more than 400 district teams directly under the supervision of local authorities spread over 33 provinces. Disease surveillance and response are the direct responsibility of district veterinary services. The PS program in Indonesia began as a pilot in 12 districts with 48 staff, collaboratively implemented by the Government of Indonesia Ministry of Agriculture, the Food and Agriculture Organization of the United Nations (FAO) and Tufts University (Jost et al. 2007), and supported by the United States Agency for International Development (USAID). It was rapidly scaled-up with the support of USAID, the Government of Japan, the Australian Agency for International Development (AusAID) and the World Bank, resulting in more than 2,000 practitioners in 31 provinces by March 2009 (Azhar et al. 2010).

#### The PS System

A rapid assessment of the HPAI situation that included poultry production and marketing practices was undertaken to develop the program design and training plan. Trainees were primarily public and private animal health practitioners (veterinarians and para-veterinarians) drawn from the district level. Initially the training involved an introductory 6-day PS course, fieldwork with supervisory visits for approximately 3 months, and then a refresher training to review and revise. In 2007, the introductory course was extended to 10 days in line with recommendations from other programs (Mariner et al. 2011a). The training covered data collection, including semi-structured interviews, proportional piling, and participatory mapping (Mariner and Paskin 2000). The PS staff carried out active HPAI surveillance in villages, identified and controlled outbreaks, trained communities on HPAI prevention and followed up on outbreak reports. Standard data collection formats were used and were analyzed centrally.

A case definition to identify and confirm HPAI outbreaks was designed collaboratively with PS practitioners based on their experience with the disease as well as PE outbreak investigations carried out throughout the island of Java in late 2005. The case definition was designed to capture disease patterns commonly observed and reported by farmers, focusing on the fact that affected flocks always exhibit cases of sudden-death, and that the disease was clearly contagious and spread between households. Outbreaks that fitted the case definition were tested using an influenza A rapid antigen test (Anigen®), and those that were positive were considered to be HPAI. The sensitivity and specificity of the PS diagnostic procedure was estimated from 128 field visits conducted in 2007 in Yogyakarta Province (Robyn et al. 2012). For outbreaks in areas where the disease had not been previously diagnosed, PCR confirmation of the diagnosis was recommended.

Although HPAI was widespread in backyard, semi-commercial and commercial producers, the PS practitioners focussed on the backyard sector due to commercial sector distrust of public interventions and ease of entrance to backyard systems. Communication materials were developed specifically for disease response, focussing on helping backyard farmers to understand how the disease spread, how to prevent and control the disease in poultry, and to prevent human exposure. The materials included flip charts, story cards, and puppets, and were based entirely on pictures with accompanying notes for the practitioner. Another innovation was to link PS officers to a program in the Ministry of Health, supported by World Health Organization, to train local public health officers to investigate and respond to HPAI outbreaks. Practitioners worked closely with human health staff when outbreaks were identified.

In May 2008, the program was refined. A protocol was implemented in which a village was considered *free* from HPAI if there had been no detections in the past 60 days, *infected* if the case definition was matched and there was a positive rapid test, *suspect* if the case definition was matched but no rapid test result was available, and *controlled* if there were no further detections in an “infected” or “suspect” village during the 60-day waiting period (Azhar et al. 2010). The case definition was redefined to capture the differences between disease patterns in backyard and commercial flocks, as well as in vaccinated and non-vaccinated birds. Whereas previously evidence of contagious disease spread had to be present, the new definition considered sudden death in one bird for backyard flocks as potentially being HPAI. In commercial flocks, unexplained mortality of over 1% was considered suspect.

#### Results

When PS for HPAI started in January 2006, the extent of the disease situation was unknown (Jost et al. 2007). During the pilot phase it was found that HPAI was widespread in the backyard sector, in addition to affecting semi-commercial and commercial producers. Multiple outbreaks were found in 11 of the 12 pilot districts within the first 3 months of the program operation. After scaling up, it was evident that HPAI was endemic throughout the country (Jost et al. 2007). Using field data and samples from 2007, the sensitivity and specificity of the PS clinical case definition utilized in the program was estimated as 89 and 95%, respectively. The sensitivity and specificity of the PS diagnostic procedure (clinical case definition integrated with an Anigen® rapid test) was 84 and 100%, respectively. The authors noted that the results gave confidence that the PS diagnostic procedure was not misdiagnosing other causes of sudden death as HPAI (Robyn et al. 2012). By 2009, HPAI had been confirmed in 31 of the country’s 33 provinces, although 86.3% of villages searched were considered apparently free. The sensitivity of Indonesia’s HPAI surveillance program was greatly enhanced with PS, and the response time to a HPAI report was reduced to 1.5 days (Azhar et al. 2010). Critical epidemiological characteristics of the disease in Indonesia, such as its geographic distribution and seasonal fluctuations in outbreaks, were determined using the PS data (ILRI 2012).

#### Lessons Learned

At the village level, PS practitioners used their communication skills to develop trust between communities and the government to encourage passive reporting and allow some control activities to take place. The link that the program tried to create between disease detection and disease response was weak, because the poorly resourced district services, who were responsible for response, were often overwhelmed by the level of disease detection achieved (Jost et al. 2007). Thus, there was wide variation in the implementation of control measures.

The PS program remained parallel to the normal national surveillance and reporting system, in that PS data was collected, transmitted and maintained separately (Azhar et al. 2010). This dramatically improved the speed and completeness of reporting at the time, but was an important constraint to the institutionalization of the program. The senior decision-makers were aware of this trade-off, but chose to instruct that the program be implemented as an emergency action outside of normal channels.

In such a large program, implemented over many years, it was a challenge to maintain the level of participation and enthusiasm of the PS staff for HPAI surveillance and control. The estimates of sensitivity and specificity found in the Yogyakarta study are probably indicative of the potential of focused, well-run programs that pay close attention to quality of training and implementation. As the Indonesia program grew, the quality of PS practice, particularly the ability to think critically and use PS as a problem-solving methodology, varied widely between areas. Due to the size of the program, quality control procedures such as mentoring visits were difficult to maintain and training messages suffered from dilution as they were relayed through the program hierarchy.

The program was expensive due to the large numbers of staff required by the decentralized design and the allowances and transport costs associated with active field work. In the later stages of the program, when community trust had been built, passive reporting increased dramatically. The PS teams investigated and confirmed passive reports, which now yielded more confirmed outbreaks than the active search activity. Control activities were difficult throughout the program due, in part, to lack of compensation for culling (Perry et al. 2009). The Perry review noted that the overall control program needed to develop solutions for issues for the commercial sector, beyond what PS could accomplish in the backyard and semi-commercial sector. This review also noted that PS, used in a risk-based manner, was not suitable for making epidemiological estimates of prevalence. However, the epidemiological objective of the surveillance program was outbreak identification for response, not estimation of epidemiological parameters. These probably could be obtained more cost effectively through targeted studies rather than as part of the surveillance system. The Perry review indicated that PS had made a very positive contribution to the animal health institutions of Indonesia and asked how such a program could best be institutionalized.

Also, it was noticed that PS practitioners, with their focus on HPAI, were losing their skills in identifying and controlling other livestock diseases. With reducing donor funding and reduced levels of HPAI in Indonesia, it was realized that the cost efficiency of the program needed be improved by including other livestock diseases. In 2012, the PS system was simplified and the operational costs reduced by focusing the program activities on response to passive reports and reducing the active search for outbreaks. Thus, one of the lessons was that PS does not need to continue ad infinitum—the system improved linkages between poultry-keepers and veterinary services so that outbreak reporting improved, and therefore PS did not need to be carried on as a routine exercise. It is best used as a time-bound, focused activity to meet specific objectives.

The Government of Indonesia has recognized the value of PE approaches in building trust between the community and the Government, and has used participatory approaches in the national veterinary service pilot program to collect information in a participatory manner; the data will be used to estimate incidence, mortality, and morbidity for selected diseases including HPAI. For example, in Bali where mass dog vaccination has been used to control a rabies epidemic, participatory surveillance will be used to search for remaining foci of rabies.

### Example 2: Sub-Saharan Africa

#### Background

In Sub-Saharan Africa, H5N1 HPAI was first reported in February 2006 in Nigeria (Kwaghe et al. 2011), and subsequently in 10 other African countries (Cattoli et al. 2009). The Early Detection, Reporting and Surveillance for Avian Influenza in Africa Project (EDRSAIA) was started in 2008 with a main objective of increasing the capacity of veterinary services in practical, community-focused, active surveillance. The project, funded by USAID, was implemented by the veterinary services of 11 countries in West and East Africa (Nigeria, Benin, Togo, Burkina Faso, Cote d’Ivoire, Liberia, Sierra Leone, Kenya, Tanzania, and Uganda) by the International Livestock Research Institute (ILRI) in collaboration with the African Union-Interafrican Bureau for Animal Resources (AU-IBAR) and Vétérinaires Sans Frontières-Belgium (VSF-B). Poultry densities and production systems varied widely between countries (FAO 2005).

#### The PS System

HPAI surveillance systems in each project country were assessed in 2008. Based on the gaps, needs and priorities were identified and national and regional stakeholders were engaged in dialog to develop a plan to build surveillance capacity focused on training in PS. Each country chose to train small PS teams of highly skilled practitioners using standardized training (Ameri et al. 2009) and performance assessment protocols. The training sequence involved a 10-day introductory training course on PE or PS, followed by a period of fieldwork that included a supervisory visit by a master trainer, and a 2–5-day refresher training to address weaknesses detected during supervision. In addition, the trainees’ supervisors received training so that all levels of the veterinary services were aware of the program and the strengths and weaknesses of the data generated. As far as possible, PS data was integrated into national reporting structures and databases. The diagnostic protocol used a clinical case definition followed by a rapid field test, and, in areas where HPAI had not been previously diagnosed, laboratory confirmation.

There had been no known HPAI outbreaks in the participating countries of East Africa, so the objective of PS in these countries was to target higher risk areas to find out whether HPAI was currently or had been recently circulating in the area. Secondary objectives were to obtain a better understanding of poultry-keepers knowledge of diseases, and the disease prevention and control measures that they apply.

#### Results

No outbreaks of HPAI were diagnosed by PS in any of the participating countries. The countries found PS to be an important surveillance tool that increased confidence that disease was in fact no longer present and that could be usefully applied to other disease control activities. It was integrated into regional Field Epidemiology and Laboratory Training Programs supported by the US-Centers for Disease Control (CDC), and has been used for additional surveillance and disease investigations. A PS team diagnosed PPR in Nigeria, which led to an effective emergency disease control program. In 2010, Nigeria chose to use PE practitioners trained by the EDRSAIA project to investigate the status of priority livestock diseases in five regions of the country (Mohamadou et al. 2011).

#### Lessons Learned

The EDRSAIA program was implemented as part of the HPAI emergency response. The objective of the surveillance was essentially set by the international pandemic response agenda that did not take a development or institution building approach. The emergency nature of the intervention led to cutting of corners and many of the best practice and previous lessons learned from early applications of PS were not implemented. For example, decision-maker workshops and other participatory activities to assess and plan national surveillance programs in partnership with stakeholders were limited. On the other hand, lessons such as having a time-bound program, clear objective, focusing on a small number of well-trained practitioners were included in the program design.

The epidemiological results of the EDRSAIA program led to a better understanding of the epidemiology and transmission risk of HPAI in relation to poultry production systems in the region, and documented the absence of endemic disease in participating countries. The results of the EDRSAIA project suggested that poultry densities (FAO 2005) were insufficient to sustain transmission, and therefore there was no need for on-going PS that targeted HPAI.

The capacity to carry out PS empowered and energized local surveillance actors. Thus, the approach is being applied to solve new problems as they arise. There is a strong interest and commitment to PS and PE throughout the region. West African countries and Uganda have formed PE networks of their own that are linked under the PENAPH umbrella, and have carried out further practitioner training courses after the completion of the EDRSAIA Project. Participatory surveillance and PE are now part of curricula in veterinary schools in West and East Africa.

### Example 3: Republic of South Sudan

#### Background

In August 2006, HPAI H5N1 was confirmed in a flock of chickens in the town of Juba, Central Equatoria State. It was suspected that the disease had been introduced from Khartoum, where an HPAI outbreak had been confirmed in April 2006, via air or boat transport of live poultry or poultry products. After the first report, all subsequent reports of poultry disease were investigated and sampled. No further cases of HPAI were detected, but two cases of Newcastle Disease (ND) were diagnosed. In order to better understand the HPAI status of Juba, it was therefore necessary to carry out active surveillance. The Ministry of Animal Resources and Fisheries (MARF) decided to use PS to investigate Juba town and the surrounding residential areas during 2 weeks in November 2006, with the support of FAO and VSF Belgium (MARF 2006).

#### The PS System

Because PS had been used in South Sudan for active rinderpest surveillance, a number of veterinarians were already experienced in its use. A 1-day workshop for 14 veterinarians and livestock officers from both Juba and other States was held to refresh practitioners’ skills and to design a semi-structured interview checklist and tools (timeline, ranking, scoring, observation of poultry) for investigating HPAI. The differences between conducting PS with cattle pastoralists and with urban poultry keepers were taken into account. The PS aimed to determine: whether HPAI was still present in the Juba area; the time-line of its occurrence over the previous year; whether ND had been present during the previous year; and whether poultry owners could tell the difference between HPAI and ND.

The veterinarians worked in pairs, with each team covering one part of the study area. They first consulted local leaders, and then conducted individual or group interviews at pharmacies, farms, and poultry-keeping households throughout the area. When suspected cases were detected, laboratory personnel were called to collect samples, conduct post-mortem examinations and HPAI rapid tests. A total of 251 group and individual interviews were conducted with 1,213 informants from approximately 600 poultry-keeping households, which was estimated to represent about 90% of all poultry-keeping households in the area. In addition, interviews were conducted with two veterinary pharmacies, two poultry sellers and four farmers with larger poultry flocks (greater than 100 birds).

#### Results

The main findings were that ND and ND-like diseases were reported to be very common at the time of the investigation, and had been occurring regularly for many years. There was no mention of any new diseases appearing in the previous 6 months. However, the level of local knowledge of poultry diseases was found to be low. Informants did not describe disease syndromes in much detail, so it is unlikely that they would have been able to differentiate between ND and HPAI if it had been present. Twelve clinical cases of poultry diseases were detected, some of which showed high mortality. These cases were sampled and all were negative for HPAI and ND on laboratory testing. Since the coverage of poultry-keeping households was high, it is likely that if HPAI had been clinically present at the time of the search it would have been detected. It was concluded that there had been no or only limited circulation of HPAI since the first outbreak. There has been no further HPAI outbreak in the Juba area since that time, which supports the PS findings.

#### Lessons Learned

One of the epidemiological lessons of the investigation was that the low poultry densities in the area were unable to maintain HPAI transmission. Similar time-bound PS was conducted in the other major towns of South Sudan and also found no HPAI. Given the epidemiology of HPAI in South Sudan, the PS approach for HPAI did not need to be sustained. South Sudan was one of the places where PS capacity had already been developed before the occurrence of HPAI, which facilitated rapid implementation of PS in response to the HPAI outbreak.

### Example 4: Egypt

#### Background

Egypt has some of the highest poultry densities on the African continent, with most birds concentrated along the River Nile. After the first Egyptian outbreaks of HPAI H5N1 in poultry in February 2006, there was widespread culling in both commercial poultry farms and household poultry. Due to the fear created by this initial response, there was limited subsequent reporting of disease outbreaks and therefore uncertainty over the frequency and distribution of HPAI, especially in the household poultry sector. Vaccination was introduced by the government in March 2006, but no significant surveillance was undertaken and from 2007 to 2008 most HPAI outbreaks in poultry were detected as part of the response to suspected or confirmed H5N1 infections in humans. Detection and reporting of poultry outbreaks was negatively perceived by some local authorities. They were concerned that detection of outbreaks would indicate animal health service inadequacies and be received as evidence of poor performance, and therefore did not always effectively respond to recognized poultry outbreaks with HPAI control measures.

In Egypt, in 2006–2007, about 40 million birds were culled and there was an apparent mismanagement of the compensation system in place. In subsequent years, the public veterinary authorities and local administration pursued the same policy of culling but without any compensation. This seriously compromised the trust between the authorities and producers and resulted in a dramatic drop in official HPAI case reporting. In 2010, FAO assisted in devising a revised HPAI control strategy where new approaches to limit spread of disease were proposed that encouraged public–private partnerships to build trust among stakeholders and enforcement of polices acceptable to producers.

In mid-2008, PE was introduced in Egypt as part of the Strengthening Avian Influenza Detection and Response (SAIDR) Project. The project was implemented by FAO and the Ministry of Agriculture and Land Reclamation (MALR) with technical input from ILRI (ILRI 2009).

#### The PS System

The first step was to conduct a rapid assessment to obtain an overview of animal health institutions, the poultry sector, HPAI surveillance and control, and the existing surveillance system to determine how PE could contribute. This was followed by a 3-day PE workshop for managers and policy-makers to introduce the key principles, methods and applications of PE, and to plan for the integration of PS into the HPAI prevention and control program so as to address priority gaps related to HPAI epidemiology and HPAI surveillance. In addition, 10 senior government veterinary personnel visited the Indonesian PS program.

The PS training program was conducted in accordance with the capacity building recommendations of PENAPH (Mariner et al. 2011a). In the first phase, 14 veterinarians from three Governorates and six veterinarians from the national General Organization of Veterinary Services (GOVS) and FAO participated in an introductory 10-day training course. They then carried out three months of HPAI PS, working in pairs to visit 1-2 high-risk villages per week, during which they received a mentoring visit from their trainer for guidance and assessment. This was followed by a refresher training, during which the HPAI PS methods and case definition for suspected HPAI were revised. A PS reporting form was developed for data entry into a national GOVS database. Suspected HPAI cases detected via PS were tested using a rapid antigen test, and reported to the Veterinary Directorate in the Governorate for collection of samples and testing at the national poultry laboratory.

In the second phase, a training of trainers’ course was conducted for 12 practitioners, who were then supported to train additional PS practitioners from other Governorates. These new practitioners did field work and received refresher training in their home governorates. In 2010, additional components of animal health communication and outbreak investigation were added and the program was renamed as Community-Based Animal Health and Outreach (CAHO). A training of CAHO trainers was conducted for 12 of the original group, who were then supported to train additional veterinarians from other Governorates.

#### Results

**Figure 1:**
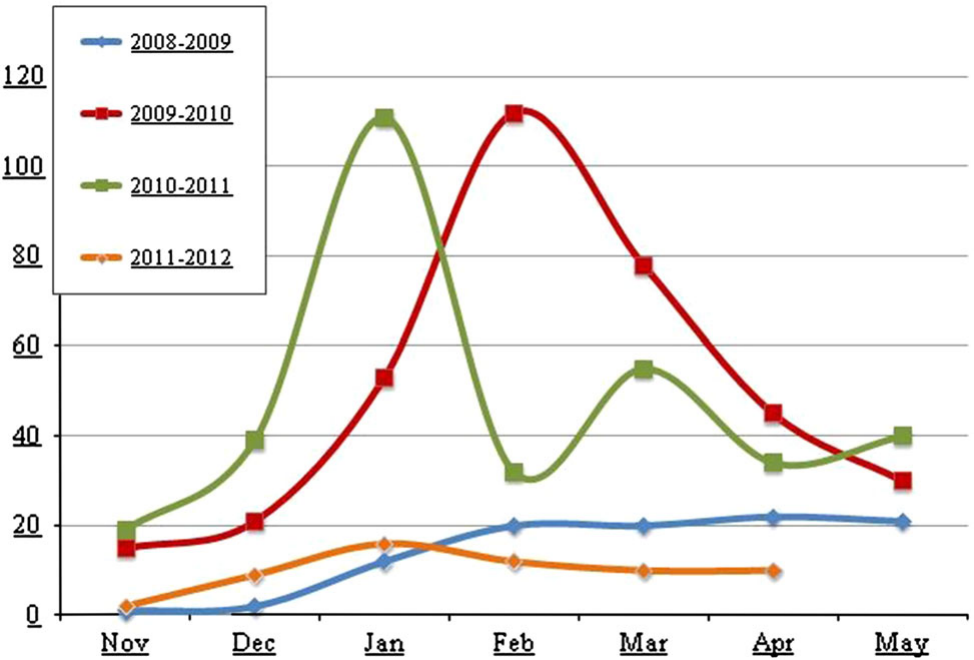
Number of annual reported A/H5N1 cases in poultry in Egypt during peak outbreak seasons (Nov–May) (2008–2012).

**Figure 2:**
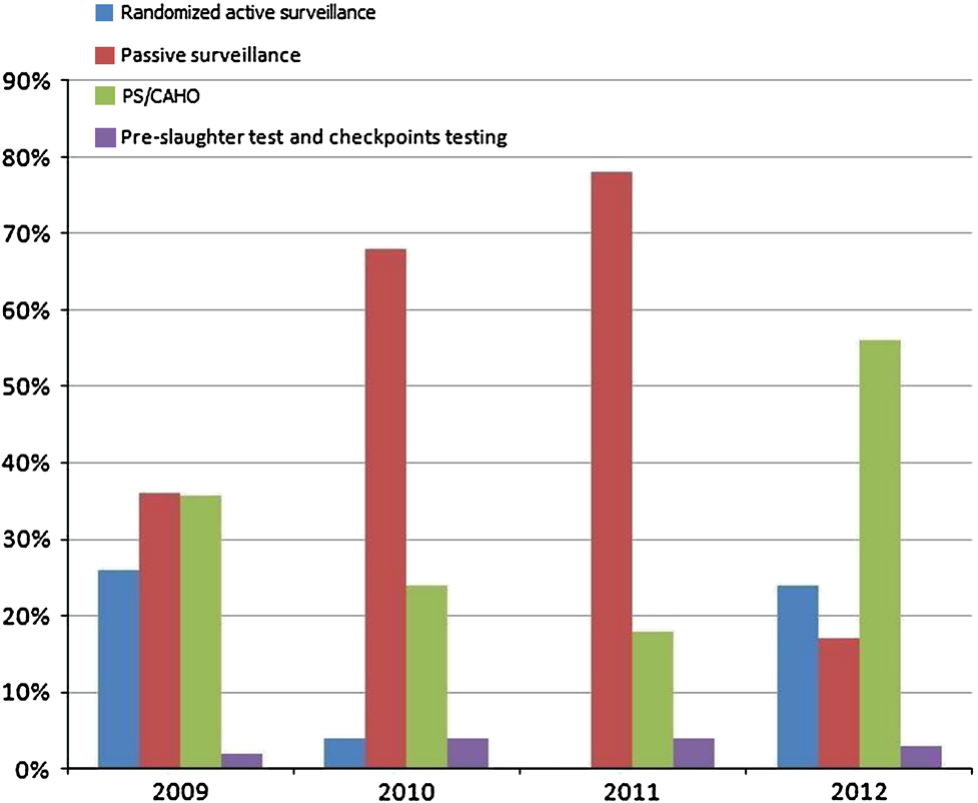
Proportion of annual A/H5N1 HPAI cases reported through the different surveillance plans from 2009 to 2012. Surveillance activities by veterinary services have slowed down since January 2011 due mainly to the socio-political changes in the country. During this period, it was noted that the CAHO surveillance program contributed over 50% of the reported HPAI cases.

The PS program was operational by December 2008, and started reporting HPAI cases in 2009 (Fig. 1). Out of 88 suspected cases that were sampled by CAHO teams in that year, 57 (65%) were confirmed positive for HPAI (Rushton and Rushton 2009). By the end of 2010, 108 veterinarians had been trained, of which 24 were national trainers and were functional in 4–6 districts in each of 15 high or medium HPAI risk governorates, out of 27 governorates. In 2011, when the overall HPAI surveillance slowed down due to the socio-political situation associated with the “Arab Spring,” the CAHO program proved to be an important tool contributing over 50% of the confirmed cases in 2012 (Fig. 2). The sensitivity and specificity at the flock level of the PS methodology based on the clinical case definition in use in Egypt was estimated as 70.1 and 83.7%, respectively (Verdugo et al. in preparation).

#### Lessons Learned

The CAHO program proved to be a robust surveillance tool for the Egyptian veterinary services, significantly contributing to the detection and reporting of HPAI outbreaks in the household and small-scale poultry production sectors and complementing the passive reporting system (Vergne et al. 2012). The study visit to Indonesia was effective for sharing lessons learned. One of the key concerns was sustainability after the end of the SAIDR project. An important decision made during the initial planning workshop was to limit the number of PS practitioners to 4–8 per Governorate and locate them only in areas where HPAI disease was considered to be an important risk. This allowed easier monitoring and quality control. In 2010, the entire CAHO program was successfully handed over to the GOVS. The CAHO program is now fully integrated into the national veterinary services. In early 2012, Egypt experienced a widespread foot-and-mouth disease (FMD) outbreak due to a new SAT2 serotype. Appreciative of the CAHO role in HPAI detection and reporting from the household and small-scale poultry production sectors, GOVS adapted the PS program to FMD surveillance with FAO providing technical assistance to reorient CAHO practitioners for the application of PS methodologies to FMD and other trans-boundary ruminant diseases.

The CAHO program faced some major challenges. The success of the teams in detecting HPAI outbreaks was not always welcomed by senior authorities in Governorates due in part to the perception that the identification of HPAI cases reflected badly on the performance of the local veterinary services. This led to a decline in case reporting and affected transparency and probably negatively affected disease control. It was recognized that such misunderstandings stemmed from inadequate consultation with stakeholders and important political decision-making powers. This challenge was mitigated by holding a series of meetings with concerned authorities.

## Discussion

The main purpose of surveillance is to inform decision-making and action in a timely manner. Surveillance programs should be designed to meet explicit objectives which in turn determine the appropriate balance of performance criteria for the system. These performance criteria generally include the sensitivity, specificity, timeliness, representativeness, simplicity, flexibility, and stakeholder ownership of the system (CDC 2001; Drewe et al. 2012). A mix of surveillance activities with different strengths can meet the requirements for effective, fit-for-purpose surveillance (Mariner et al. 2011a).

Experience has shown that surveillance programs often lack the necessary levels of sensitivity and timeliness required to inform effective response. In the case examples where HPAI was present, PS improved case finding and the speed of reporting. In the case examples where HPAI was not present, PS did not lead to false positive reports or unnecessary alarms. Without PS, disease problems often go undetected or are severely under-reported and this was the experience in HPAI control. These gaps are related to issues of bias in the methods applied and the institutions responsible for implementing surveillance. For example, small-scale and backyard production systems are often under-represented in comparison to commercial agriculture. Or, pastoral areas that lack animal health infra-structure, do not have effective networks for collection and communication of surveillance data. In addition, hierarchical reporting systems often filter out considerable amounts of information due to the different incentive structures for personnel and managers at various levels of the system. This leads to a surveillance database that only reflects a small proportion of the information available at the grass-roots level and that may actually be misleading.

The success of surveillance activities is related to the incentives that motivate participants to contribute information to the system and for surveillance actors to support the transmission of information along the chain. The term “incentive” encompasses all the reasons why people make choices. The incentives that drive stakeholder behavior in surveillance systems include the economic, social, psychological, and institutional outcomes that will result from their choices or actions. If, on balance, the action results in a positive outcome for the participants, they are more likely to report. On the other hand, if the outcome is largely negative, they are unlikely to contribute.

Surveillance participants (practitioners and livestock owners) involved in PS program have indicated that the following positive incentives improve the performance of surveillance:

- Easy access to the surveillance system that recognizes the value of people’s time
- Surveillance personnel show genuine respect for knowledge and information provided
- Surveillance personnel seek to build trust
- Surveillance information leads to action that has a positive impact at the household level or at the level of the reporter

Examples of negative incentives identified by surveillance participants include:

- Time and money invested in providing surveillance information rarely result in action
- Providing surveillance information results in actions that negatively affect household or community well-being
- Disease reporting result in social isolation of the participant or community retribution
- Transmission of disease reports is perceived to endanger the careers of surveillance actors or place informants in stress situations

Altruistic considerations such as “it’s the right thing to do” can be a positive incentive, but for most surveillance actors this is probably not a major driver of choices. Mixed messaging in health institutions often contributes to weak performance of surveillance systems. For example, managers may circulate instructions that all outbreaks should be reported and then react negatively to specific reports. Local departments may be under pressure to show positive performance and reports of disease outbreaks may be perceived as evidence to the contrary, as was the case in Egypt. Response strategies that rely heavily on market closure, quarantine, or culling often lead to insensitive surveillance systems. Certainly, the HPAI culling experiences in Egypt and Indonesia and subsequent reluctance of poultry owners and professionals to report cases illustrate this point (UNICEF 2010).

In the HPAI case examples, the implementation of PS introduced positive incentives for reporting and mitigated several negative incentives. Farmers experienced easier access to surveillance systems and the approach made them feel that their information and knowledge was valued. Surveillance personnel were energized by the new knowledge they gained and the new relationships that they were able to form with producers. In both Indonesia and Egypt, PS worked to mitigate the negative perceptions of the outcome of reporting and control actions created by the initial mass culls.

Previously, PS has been used as a risk-based, purposive approach to surveillance that qualitatively targets populations with high transmission risk who may act as endemic reservoirs for infection. In applications prior to HPAI and in the HPAI programs, PS usually found more outbreaks of disease than any other surveillance method applied, when disease was present in the country. This included a randomized pre-slaughter sampling and testing program conducted at high expense in Egypt (Rushton and Rushton 2009; Yrjö-Koskinen et al. 2010). Only 8 of 24,253 (0.03%) samples were positive in the pre-slaughter survey whereas 57 of the 88 (64.77%) samples submitted by PS teams were positive. In Nigeria, a similar approach to laboratory-testing-based active surveillance utilizing a national randomized sample yielded no positive results (Oladokun et al. 2012). Initially, in Indonesia, active searching using PS resulted in the vast majority of disease detections. However, once the program was well established, passive reporting into the PS system became the major source of leads for investigation.

The Indonesian example was by far the largest PS program ever undertaken. At the outset, the incidence of outbreaks was entirely unknown and the program design called for outbreak search and containment. It rapidly became apparent that HPAI was a common disease in Indonesia and the numbers of outbreaks detected on a weekly basis by the end of year one overwhelmed a case-by-case response plan. However, this knowledge probably could have been obtained in a more cost-effective manner by a limited number of PS teams taking a periodic epidemiologic assessment approach rather than using an on-going surveillance model. In hindsight, the surveillance and control models should have been changed much earlier as soon as the PS data indicated that HPAI was frequent and widespread.

The application of PS to HPAI involved the development of a syndromic case definition for sudden death and an HPAI specific case definition in all programs. Poultry owner knowledge emphasized that sudden death (a clinical illness with death occurring within minutes to 3 or 4 h from the onset of detectable symptoms) was the principle distinguishing factor of HPAI outbreaks. The availability of rapid tests was a major advantage and all PS programs were advised to give rapid tests to all PS teams. Some countries resisted this recommendation. The results of sensitivity and specificity analysis from Indonesia and Egypt document the performance of two different clinical PS procedures for HPAI in different epidemiological context. When a rapid test was integrated in the Indonesia procedure, sensitivity decreased slightly from 86 to 84% and specificity increased form 94 to 100%. This illustrates the impact that incorporation of a rapid test can have on the program.

Hannah et al (2012) have noted an absence of institutionalization of PS activities, but apparently utilized as an indicator the existence of standardized procedures and structured surveillance programs as are very common in public health surveillance systems. However, in the countries considered, there is strong evidence of a committed community of practitioners, local adaptation of techniques, application of the techniques to subsequent problems, peer-reviewed publications on multiple issues led by national stakeholders (for example Anjum et al. 2006; Hussain et al. 2008) and incorporation of the techniques in curriculum for professional training in national Universities. As has been noted, the strengths of PS are founded on the flexible, problem-solving methodologies of PRA geared towards discovery and expansion of perceptions. Each application of PS should be tailored to specific objectives and have a beginning and an end. The authors of this paper argue that the most important contribution that PS can make is through adding new dimensions to surveillance. We discourage regimented approaches and believe that PS practitioners should be able to adapt interview checklists and select the correct mix of tools suited to the situation and their own communication styles.

When comparing PS for HPAI to other PS experiences, both the nature of the disease and the species affected is a consideration. Most applications of PS have dealt with diseases that have distinguishing features, which are readily recognized by the livestock owners, and addressed disease problems with significant economic impact in high value species. HPAI does not have pathognomonic features and its impact stems from both its livelihood and public health importance. However, the threat of a global pandemic as perceived by the international health community was not a principle driver for disease reporting for most actors in the developing world. A few key issues stand out.

- The sudden death syndrome was remarkable, noted by livestock owners and associated with HPAI.
- Poultry owners value backyard poultry keeping as an activity but assume significant mortality will occur and that the populations will naturally regenerate without major intervention.
- The response to disease reports often had a negative impact, which was a disincentive for farmers to report disease, or for district or central government to pass on reports.

No two applications of PS are the same. It is a flexible methodology that can be adapted to technical and social issues associated with specific disease problems. Although not a panacea, PS detected HPAI effectively and accurately (Robyn et al. 2012; Verdugo et al. in preparation), characterized its epidemiology and contributed to mitigating many of the social constraints to effective surveillance and control associated with such a politically significant zoonosis.

## Lessons

These experiences with PS for HPAI surveillance, as well as two decades of prior experience with a variety of livestock diseases on three continents, have provided many lessons in how best to integrate PS into surveillance systems. The authors wish to highlight ten very important lessons that should be taken into account in the design of future programs.

- Targeted disease searches depend on a clinical case definition, which needs to be fit-for-purpose, well articulated and consistently applied by all practitioners.
- Decision-makers need to understand the nature of a PS diagnosis and have a clear understanding of the accuracy of the diagnostic procedure used in a program. For this, as well as a variety of other reasons, the training of decision-makers, particularly the supervisors of PS practitioners, is critical.
- A surveillance system must have a well-defined objective to guide the design of the system and the specification of the activities and types of data necessary to meet that objective. Otherwise, the system risks being a group of disparate activities that result in data documentation but inconsistent action.
- A surveillance system must be appropriately budgeted and resourced in terms of people, capacity, and materials. This includes resources that are necessary for active outreach such as PS, which can push budgets beyond what had been planned in the past. In such a system, PS inherently becomes one component of a comprehensive system (Mariner et al. 2011a).
- For PS to succeed as a surveillance tool, the most important consideration is the quality of the PS practitioners, not the number or size of the program. Thus, training is the key and this must include a standardized training program and protocol for continual mentoring and evaluation of practitioners. It is important to create sustainable capacity within veterinary services, private animal health systems, and academia.
- Participatory surveillance is a problem solving methodology. Programs that focus on creating a small cadre of well trained individuals capable of carrying out assessments that provide accurate best-bet scenarios to inform policy is perhaps the best application of the method.
- Training to create capacity to search for one disease is self-limiting and not intended to be sustained. Focusing on key concepts, appropriate use of tools and analysis, and flexibility will help a veterinary service meet current threats, as well as give them tools to respond to future problems.
- It is critical that data generated by PS is reported using normal national channels and structures, and that data is integrated into national disease databases. Combined with policies that recognize the complementarity of PS data, PS thus becomes an additional disease reporting tool for veterinary service.
- Participatory surveillance is a risk-based surveillance tool. The World Animal Health Organization (OIE) risk analysis framework emphasizes the need to link risk assessment and management. Surveillance does this, as it generates the data necessary for risk assessment and management actions such as disease prevention and control.
- Any surveillance program, as well as each of its components, should be tied to a disease control plan. That plan must include a review of the policies, resources, and culture that govern disease control actions, so that an enabling atmosphere is in existence prior to detection of a disease threat.

Finally, veterinary services that have adopted PS as a surveillance tool have often found that the new information gained through the perspective of livestock owners encourages them to reconsider current policies, particularly concerning surveillance design and disease prioritizations. This creates an opportunity to improve services through institutional change to meet the needs and expectations of livestock owners as critical stakeholders.

## Conclusion

The use of PS for HPAI surveillance was widely received as a positive experience that enhanced surveillance, and was rewarding for those involved in delivering the programs. Small programs with a focus on creating a nucleus of highly competent individuals capture most of the benefit of the approach and are economically more sustainable than large programs. In the countries and regions where it was applied to HPAI, PS has been adopted and adapted to national needs and is now part of the institutional landscape. Beyond the positive effects that PS training can have on human capacity and organizational culture, its best use is as a purposive tool to address specific surveillance objectives formulated to address the challenges of the future.

## References

[CR1] Admassu B (2005) The participatory epidemiological investigation of FMD in Erzurum Province. www.participatoryepidemiology.info/userfiles/Admassu-FAO-Turkey-PE.pdf. Accessed 4 Oct 2009

[CR2] Ameri AA, Hendrickx S, Jones B, Mariner J, Mehta P, Pissang C (2009). Introduction to participatory epidemiology and its application to highly pathogenic avian influenza participatory disease surveillance. A manual for participatory disease surveillance practitioners.

[CR35] Anjum R, Hussain M, Zahoor AB, Farooq HIU (2006). Epidemiological analyses of foot and mouth disease in Pakistan. International Journal of Agriculture and Biology.

[CR3] Azhar M, Lubis AS, Siregar ES, Alders RG, Brum E, McGrane J, Morgan I et al (2010) Participatory disease surveillance and response in Indonesia: strengthening veterinary services and empowering communities to prevent and control highly pathogenic avian influenza. http://www.aaapjournals.info/doi/abs/10.1637/8713-031809-Reg.1. Accessed 7 Nov 2012 10.1637/8713-031809-Reg.1 20521726

[CR4] Bett B, Jost C, Allport R, Mariner J (2009). Using participatory epidemiological techniques to estimate the relative incidence and impact on livelihoods of livestock diseases amongst nomadic pastoralists in Turkana South District, Kenya. Preventive Veterinary Medicine.

[CR5] Catley A, Alders RG, Wood JLN (2012). Participatory epidemiology: approaches, methods, experiences. Veterinary Journal.

[CR6] Catley A, Chibunda RT, Ranga E, Makungu S, Magayane FT, Magoma G, Madege MJ, Vosloo W (2004). Participatory diagnosis of a heat-intolerance syndrome in cattle in Tanzania and association with foot-and-mouth disease. Preventive Veterinary Medicine.

[CR7] Catley A, Okoth S, Osman J, Fison T, Njiru Z, Mwangi J, Jones BA, Leyland TJ (2001). Participatory diagnosis of a chronic wasting disease in cattle in southern Sudan. Preventive Veterinary Medicine.

[CR8] Cattoli G, Monne I, Fusaro A, Joannis TM, Lombin LH, Aly MM, Arafa AS (2009). Highly pathogenic avian influenza subtype H5N1 in Africa: a comprehensive phylogenetic analysis and molecular characterization of isolates. PLoS One.

[CR9] CDC (2001). Updated guidelines for evaluating public health surveillance systems. MMWR.

[CR10] Chambers R (2007) From PRA to PLA and pluralism: practice and theory. IDS Working Paper 286, July 2007. http://www.ids.ac.uk/publication/from-pra-to-pla-to-pluralism-practice-and-theory. Accessed 18 Feb 2014

[CR11] Drewe JA, Hoinville LJ, Cook AJC, Floyd T, Stärk KDC (2012). Evaluation of animal and public health surveillance systems: a systematic review. Epidemiology and Infection.

[CR12] FAO (2005) Predicted global poultry density, http://www.fao.org/geonetwork/srv/en/metadata.show?id=12720&currTab=distribution. Accessed 10 Aug 2013

[CR14] Hannah H, Pali P, Rware H, Bett B, Randolph T, Grace D, Njuki J, Pissang C, Hisrich E (2012) Participatory disease surveillance evaluation: findings from Africa (HPAI) & Pakistan (rinderpest). Poster presented at international symposium of veterinary epidemiology and economics 13, Maastrict, Netherlands

[CR15] Hussain M, Afzal M, Ali Q, Taylor W, Mariner J, Roeder P (2008). The epidemiology of peste des petits ruminants in Pakistan and possible control policies. Revue Scientifique et Technique.

[CR16] ILRI (2009) Introduction of Participatory Epidemiology to strengthen animal disease surveillance and control. Final Report, February 2009. In: International Livestock Research Institute, Nairobi for Strengthening AI Detection and Response (SAIDR) Project of FAO ECTAD Egypt

[CR17] ILRI (2012) Operational research in indonesia for more effective control of highly pathogenic avian influenza. Final Report for USAID Project Cooperative Agreement No. 497-A-00-07-00021-00 (p. 163). International Livestock Research Institute, Nairobi

[CR18] Jost CC, Mariner JC, Roeder PL, Sawitri E, Macgregor-Skinner GJ (2007) Participatory epidemiology in disease surveillance and research. *Revue Scientifique et Technique* 26(3):537–49. http://www.ncbi.nlm.nih.gov/pubmed/18293603 18293603

[CR19] Jost CC, Nzietchueng S, Kihu S, Bett B, Njogu G, Swai ES, Mariner JC (2010). Epidemiological assessment of the Rift Valley fever outbreak in Kenya and Tanzania in 2006 and 2007. American Journal of Tropical Medicine and Hygiene.

[CR13] Kihu SM, Njagi LM, Njogu GN, Kamande JN, Gitao CG (2012). Peste des petits ruminants in Kenya; pastoralist knowledge of the disease in goats in Samburu and Baringo Counties. Research Opinions in Animal and Veterinary Sciences.

[CR20] Kwaghe AV, Ndahi MD, Usman JG, Sambo E, Waziri I, El-Oji A, Pam EG (2011). Highly pathogenic avian influenza participatory disease surveillance in Plateau State, Nigeria. Archives Des Sciences.

[CR21] Lam TTY, Hon CC, Pybus OG, Kosakovsky Pond SL, Wong RTY, Yip CW, Zeng F (2008). Evolutionary and transmission dynamics of reassortant H5N1 influenza virus in Indonesia. (E. C. Holmes, Ed.). PLoS pathogens.

[CR22] MARF (2006) Participatory disease search for highly pathogenic avian influenza Juba, Southern Sudan, November 2006. Ministry of Animal Resources and Fisheries, Government of Southern Sudan

[CR23] Mariner JC, Catley A, Zepeda C (2002) The role of community-based programs and participatory epidemiology in disease surveillance and international trade. In: Primary Animal Health Care in the 21st Century: shaping the rules, policies and institutions. International conference Mombasa, Kenya, 15–18th Oct. http://sites.tufts.edu/capeipst/files/2011/03/Mariner-et-al-Mombasa.pdf

[CR24] Mariner JC, Manzoor Hussain, Roeder PL and Catley A (2003) The use of participatory disease searching as a form of active disease searching in Pakistan for rinderpest and more. In: Proceedings of the 10th international symposium on veterinary epidemiology and economics, Vina del Mar, Chile, 17–21 Nov 2003

[CR25] Mariner JC, Hendrickx S, Pfeiffer DU, Costard S, Knopf L, Okuthe S, Chibeu D, Parmley J, Musenero M, Pisang C, Zingeser J, Jones B, Syed Noman Ali, Bett B, McLaws,M, Unger F, Aluma Araba, Purvi Mehta, Jost CC (2011a) Integration of participatory approaches into surveillance systems. *Revue Scientifique et Technique* 30:653–659 10.20506/rst.30.3.2065 22435179

[CR26] Mariner JC, Paskin R (2000) Participatory epidemiology: methods for the collection of action-oriented epidemiological intelligence, FAO Manual No. 10, FAO, Rome

[CR27] Mariner J, Ruiz de los Rios P, Eulert Mendoza E, Lubroth J (2011b) Epidemiologia participativa, FAO Manual No. 10, FAO, Rome

[CR28] Mariner J, Pissang C, Allport R, Soumare B, Münstermann S, Parmley J, Pfeiffer D, Bloland P, Busuulwa M (2012) Update on the participatory epidemiology network for Animal and Public Health (PENAPH). Keynote presentation for the mini-symposium on participatory epidemiology. In: Proceedings of the international symposium on veterinary epidemiology and economics 13, Maastricht, 20–24 Aug 2012

[CR29] Mariner JC, Roeder PL (2003). The use of participatory epidemiology to study the persistence of Lineage 2 rinderpest virus in East Africa. Veterinary Record.

[CR30] Mohamadou F, Jost C, Ihedioha J (2011) Financial costs of disease burden, morbidity and mortality from priority livestock diseases in Nigeria. Nigeria Integrated Animal and Human Health Project. International Livestock Research Institute, Nairobi

[CR31] Oladokun AT, Meseko CA, Ighodalo E, John B, Ekong PS (2012) Effect of intervention on the control of highly pathogenic avian influenza in Nigeria. *Pan**African**Medical Journal* 13:14 PMC3527058 23308319

[CR32] PENAPH (2013) www.penaph.net. Accessed 5 Aug 2013

[CR33] Perry B, Kamarudin I, Tarazona C (2009). Independent evaluation of FAO’s participatory disease surveillance and response program in Indonesia.

[CR34] Robyn M, Priyono W, Kim L, Brum E (2012). Diagnostic sensitivity and specificity of a participatory disease surveillance method for highly pathogenic avian influenza in household chicken flocks in Indonesia. Avian Diseases.

[CR36] Rushton J, Rushton R (2009). Evaluation of the process of PE/ introduction and impact of the PDS methodology on the national surveillance system in Egypt.

[CR37] UNICEF (2010) Joint United Nations Assessment of Government of Egypt H5N1 Control Efforts, UNICEF, 6–16 Dec 2009

[CR38] Vergne T, Grosbois V, Jobre Y, Saad A, Abd El Nabi A, Galal S, Kalifa M, Abd El Kader S, Dauphin G, Roger F, Lubroth J, Peyre M (2012) EID Journal Avian Influenza Vaccination of Poultry and Passive Case Reporting, Egypt vol 18, Number 12–December 2012. http://wwwnc.cdc.gov/eid/article/18/12/12-0616_article.htm#suggestedcitation 10.3201/eid1812.120616 PMC3557869 23171740

[CR39] Verdugo C, ElMasry I, Yilma J, Hannah H, Jewell C, Unger F, Soliman M, Galal S, Lubroth J, Grace D (in preparation) A Bayesian sensitivity and specificity estimation of the participatory disease surveillance program for highly pathogenic avian influenza in Egypt 10.1637/11442-060316-Reg 27902900

[CR40] Yrjö-Koskinen A, Abascal RO, Rushton J (2010) Cost-effectiveness of HPAI surveillance systems in Egypt—final report. ILRI, Nairobi

